# Interpersonal Problems as Mediators of the Association Between Personality Disorders and Mental Health

**DOI:** 10.1002/cpp.70307

**Published:** 2026-07-16

**Authors:** L. Macía, G. Aonso‐Diego, A. Estévez, C. Mirapeix

**Affiliations:** ^1^ Department of Psychology, Faculty of Health Sciences University of Deusto; ^2^ Fundación para la Investigación en Psicoterapia y Personalidad (FUNDIPP) Santander Spain

**Keywords:** gender, interpersonal problems, mental health, personality disorders, psychopathology

## Abstract

Impairments in interpersonal functioning are a core transdiagnostic feature of personality disorders (PDs). While interpersonal problems are known to worsen clinical outcomes, their mediating role between PDs and mental health requires further empirical examination. The study aims were: (1) to analyse gender differences in PDs, mental health and interpersonal problems; (2) to study the correlations between study variables; and (3) to examine the mediating role of interpersonal problems in the relationship between PDs and global mental health. The final sample comprised 291 participants (*M*_age_ = 38.30, SD = 13.80; 66.3% women) from a clinical population who completed the International Personality Disorder Examination (IPDE), the Inventory of Interpersonal Problems (IIP‐32) and the Symptom Checklist (SCL‐90). Women scored higher on the self‐sacrificing dimension (*p* = 0.023), and a significant gender difference was also found for narcissistic personality disorder, with men scoring slightly higher than women (*p* = 0.048). Higher scores on PDs and greater interpersonal difficulties were strongly associated with mental health problems (*ρ*‐values between 0.467 and 0.571). Finally, interpersonal problems mediated the association between personality cluster scores and mental health problems. Interpersonal problems, PDs and mental health symptoms form an interlinked pattern of psychological distress. Consequently, therapeutic interventions for PDs must prioritize the modification of maladaptive relational styles with the aim of preventing or mitigating co‐occurring mental health problems.

## Summary

- Interpersonal problems play a key role in the relationship between personality disorders and mental health symptoms, highlighting the importance of assessing relational functioning in routine clinical practice.
- Difficulties such as social inhibition, non‐assertiveness, excessive accommodation, interpersonal dependency and relational distancing may contribute to the worsening of mental health problems across personality disorders.
- Clinicians should prioritize evidence‐based therapeutic frameworks that include specific modules for relational effectiveness and interpersonal dynamics (e.g., DBT and MBT).
- Clinicians should pay particular attention to self‐sacrificing interpersonal styles, especially among women, given their potential association with increased psychological symptomatology.

Personality disorders (PDs) are a group of mental disorders characterized by stable and persistent dysfunctional patterns in areas such as cognition, affective regulation, mentalization, interpersonal relationships and/or impulse control, causing clinically significant personal distress (American Psychiatric Association [APA] 2022). PDs constitute a major public health concern due to the associated functional and social impairment, as well as the extensive utilization of mental and general healthcare services (Massaal‐van der Ree et al. 2022; Skodol et al. 2005).

The revised fifth edition of the Diagnostic and Statistical Manual of Mental Disorders (DSM‐5‐TR; APA 2022) classifies PDs into three main clusters: Cluster A (the eccentric cluster) includes paranoid, schizoid and schizotypal PDs; Cluster B (the dramatic/emotional/erratic cluster) includes borderline, narcissistic, antisocial and histrionic PDs; and Cluster C (the anxious/fearful cluster) includes avoidant, dependent and obsessive‐compulsive PDs. This categorization is widely used in clinical practice, research and treatment programmes for PDs (Skodol et al. 2011; van den End et al. 2024; Winsper et al. 2020).

Although this cluster‐based classification provides a valuable and practical framework, current literature highlights that to fully capture the complexity and symptomatic overlap of these clinical conditions, it is essential to complement it by evaluating underlying dimensional variables (Herpertz et al. 2017; Hopwood et al. 2018). A consistent empirical finding is that impairments in identity and interpersonal functioning constitute core transdiagnostic vulnerability factors across all PD clusters (A, B and C) (Herpertz et al. 2017; World Health Organization [WHO] 2019). Indeed, the marked difficulty in establishing and maintaining stable, reciprocal and adaptive interpersonal relationships is not merely a consequence of PDs, but rather one of their fundamental defining features (d'Huart et al. 2023).

In this regard, it has been found that while Cluster A individuals tend towards detachment or suspiciousness in interpersonal relationships (APA 2022; Noel et al. 2023), individuals belonging to Cluster B tend to exhibit relational dynamics that are labile and unstable, chaotic or dependent on external validation. In fact, many of these relationships may alternate between idealization and devaluation (Bohus et al. 2021). Regarding Cluster C, individuals in this group tend to demonstrate greater social avoidance or inhibition (Solomonov et al. 2020) or excessive submissiveness, mostly driven by fear of rejection. In all cases, the lack of adaptation and the significant deterioration of the interpersonal and social sphere constitute one of the main predictors of chronicity, severity, poor therapeutic adherence and poorer overall quality of life (APA 2022; WHO 2019). Therefore, this transdiagnostic perspective places interpersonal problems as one of the central evaluative axes for determining the severity and diagnosis of PDs.

In line with the above, several studies show that interpersonal problems, especially those that become chronic, have a direct impact on general mental health, exacerbating and worsening the clinical symptomatology comorbid with PDs (Yiu et al. 2022; Zimmermann et al. 2015). Empirical literature demonstrates that PDs, combined with difficulties in the social sphere, correlate strongly with greater mental health problems, including elevated levels of anxiety and depressive symptomatology (Noel et al. 2023). Furthermore, the deterioration of the interpersonal dimension could complicate the clinical picture and increase vulnerability to poorer mental health (Cain et al. 2021; Laverdière et al. 2019). Given this close interrelation, recent research has begun to suggest that the impact of PDs on mental health may be mediated by the quality and functionality of social interactions (Euler et al. 2021; Lee et al. 2001; Yiu et al. 2022).

Various studies have evidenced that interpersonal problems act as a significant mediating variable between psychopathological personality traits and adverse clinical outcomes, observable across a wide variety of PDs. In this sense, interpersonal problems have been observed to mediate the relationship between different facets of PDs and anxiety (Noel et al. 2023), as well as general psychosocial impairment in patients with borderline (Cluster B) and avoidant (Cluster C) profiles (McCloskey et al. 2021). For their part, in conditions characterized by predominantly externalizing and oppositional profiles, such as narcissistic PD (Cluster B), research shows that severe interpersonal conflicts, such as dominant and low‐empathy attitudes, could act as specific risk factors for the development of depressive symptomatology and acute distress, aggressiveness, conflict‐seeking and violent attitudes, especially when the individual experiences rejection or a lack of external validation (Ogrodniczuk et al. 2009; Wright et al. 2017). Similarly, in anxious‐fearful spectrum disorders (Cluster C), such as dependent or obsessive‐compulsive personalities, maladaptive relational styles often manifest through attitudes such as extreme submissiveness, self‐sacrifice or, conversely, excessive coldness and affective relational distancing. It has been found that these types of relational attitudes could explain the subsequent development of severe anxiety, ruminative thoughts, emotional disconnection, guilt or isolation, as well as a poorer response to psychiatric and psychological treatments (Berghout et al. 2012; Wilson et al. 2017; Zimmermann et al. 2015). Regarding Cluster A PDs, which encompass paranoid, schizoid and schizotypal personalities, the literature indicates that relational styles marked by suspiciousness, difficulties in establishing intimate bonds, mistrust and extreme social disconnection are closely linked to clinical and emotional distress, such as social anxiety, hostility, interpersonal alienation, hypervigilance or depressive episodes (Derogatis et al. 1976; Esterberg et al. 2010; Mann et al. 2008).

However, it is important to consider the role of individual differences in the expression of these dynamics. The literature indicates that interpersonal profiles and the prevalence and severity of certain PDs can vary significantly, with heterogeneity in how these conditions manifest depending on sociodemographic variables such as gender. Previous research suggests that, while men tend to exhibit interpersonal profiles more strongly characterized by antagonism, hostility, detachment or emotional disengagement, even presenting a higher prevalence of antisocial or narcissistic traits, women tend to present higher scores in dimensions of emotional lability, dependence, submissiveness, somatization, self‐discipline and attitudes of relational self‐sacrifice (Christiansen et al. 2022; Paris 2004). Along these lines, other studies note that women consistently tend to score higher in neuroticism, experiencing greater emotional lability, vulnerability to anxiety, lack of self‐esteem and internalizing symptomatology, as well as in the trait of agreeableness, showing more empathetic and cooperative profiles (Hartung and Lefler 2019). Conversely, men usually score higher in facets related to assertiveness, sensation‐seeking, agency, anger, impulsivity and externalizing symptomatology (Bozzatello et al. 2024; Weisberg et al. 2011).

Despite the accumulated evidence regarding interpersonal impairment in PDs, the existing empirical literature presents limitations. First, the vast majority of research analysing the mediating role of interpersonal problems has focused on isolation in specific disorders, predominantly borderline PD (Dixon‐Gordon et al. 2011; Paris 2008) or has been limited to studying the specific profiles of Clusters B or C (Monaghan and Bizumic 2023). Therefore, vulnerability variables and how the different PDs operate need to be studied as a whole, rather than in a fragmented manner. Second, although marked gender differences in relational dynamics and the prevalence of PDs are traditionally assumed, contemporary research warns that some of these differences may be due to gender biases (Özel et al. 2025). There is therefore a need to explore potential gender‐based differences in manifestations (Samuel and Widiger 2009). Furthermore, it is worth noting that research utilizing clinical samples often relies on small sample sizes.

Therefore, the study's aims were: (1) to analyse gender differences in PDs, mental health and interpersonal problems; (2) to study the correlations between study variables; and (3) to examine the mediating role of interpersonal problems in the relationship between PDs and global mental health.

## 1 Methods

### 1.1 Participants and Procedure

A total of 809 individuals initially took part in the study. However, a substantial proportion of participants did not complete the main assessment instruments: 364 did not complete the International Personality Disorder Examination (IPDE), 384 did not complete the Symptom Checklist (SCL) and 461 did not complete the Inventory of Interpersonal Problems (IIP‐32). Given this level of missing data, imputation techniques could not be applied, resulting in a final sample of 291 participants with complete data.

The final sample (*N* = 291) had a mean age of 38.30 years (SD = 13.80), ranging from 18 to 76 years. Women represented 66.32% of the sample (*n* = 193) and men 33.68% (*n* = 98). Although on average the men (*M* = 19.98, SD = 1.51) were younger than the women (*M* = 39.36, SD = 1.05), this difference did not reach statistical significance (*p* = 0.085).

All participants were patients at a specialized centre in Santander (Spain), where they received treatment from psychologists and psychiatrists. Recruitment took place between 2010 and 2021. The clinical data utilized in this study were collected within an outpatient psychological treatment programme. The psychotherapeutic interventions were delivered by a team of psychologists (*n* = 8–9). Regarding the treatment format, the intervention consisted predominantly of individual therapy, although adjunctive family sessions were incorporated when clinically indicated. Contingent upon the patient's clinical progress, the sessions were subsequently spaced to an every‐2‐week frequency until discharge, followed by post‐treatment follow‐up sessions at one and 3 months. Importantly for the present study, all psychometric evaluations and clinical data analysed herein were collected strictly at baseline (i.e., prior to the onset of the psychotherapeutic intervention).

Moreover, prior to participation, all individuals were informed of the study objectives and procedures, and written informed consent was obtained. Participation was voluntary, with no incentives provided and respondents were assured that their decision would not affect their treatment. Anonymity and confidentiality of responses were strictly maintained. The study was approved by the corresponding Ethics Committee and conducted in accordance with the ethical standards set out in the Declaration of Helsinki.

### 1.2 Instruments

The International Personality Disorder Examination (IPDE) (Loranger et al. 1994), in its Spanish validation (López‐Ibor et al. 1996), was employed to assess the presence of PDS according to International Classification of Diseases (ICD) and DSM‐5 criteria. The IPDE comprises a total of 77 items designed to evaluate 10 specific PDs: paranoid, schizoid, schizotypal, histrionic, narcissistic, borderline, antisocial, dependent, avoidant and obsessive‐compulsive. Each subscale has 7–8 items, except for the Borderline, Schizotypal and Narcissistic subscales, which each include 9 items. In the IPDE, participants rate each item as ‘true’ or ‘false’. Some items are worded in the opposite direction, and the questions are presented in mixed order instead of being grouped by disorder.

The Inventory of Interpersonal Problems‐32 (IIP‐32) (Horowitz et al. 1988) was used to assess participants' self‐reported difficulties in interpersonal relationships. In the present study, we employed the abbreviated 32‐item version (Barkham et al. 1996), adapted and validated for Spanish‐speaking populations by Salazar et al. (2010). Items are grouped into eight dimensions that capture common interpersonal problem patterns: (1) Domineering/Controlling; (2) Intrusive/Needy; (3) Self‐Sacrificing; (4) Overly Accommodating; (5) Nonassertive; (6) Socially Inhibited; (7) Cold/Distant; and (8) Vindictive/Self‐Centred. Respondents rate the presence of each item's content on a 5‐point Likert scale ranging from 0 (*not at all*) to 4 (*very much*).

The Symptom Checklist‐90 (SCL‐90) was used to assess psychological symptomatology. This instrument was originally developed by Derogatis et al. (1976) and validated for Spanish‐speaking populations by González de la Rivera et al. (1989). The SCL‐90 consists of 90 items, each rated on a 5‐point Likert scale ranging from 0 (*no symptom‐related distress*) to 4 (*maximum distress*), indicating the degree of distress experienced for each symptom over the past week. The items are grouped into nine dimensions: (1) Somatization, (2) Obsession‐Compulsion, (3) Interpersonal Sensitivity, (4) Depression, (5) Anxiety, (6) Hostility, (7) Phobic Anxiety, (8) Paranoid Ideation and (9) Psychoticism.

### 1.3 Data Analysis

Prior to hypothesis testing, the distribution of all continuous study variables was examined. The variables included PD traits, interpersonal problems and the nine mental health symptom dimensions. Normality was tested using the Kolmogorov–Smirnov test. Results indicated that none of the variables met the assumption of normality, with all *p*‐values ≤ 0.004. Accordingly, nonparametric tests were used in subsequent analyses.

First, gender differences were explored using the Mann–Whitney *U* test. Effect sizes for significant group differences were calculated using Rosenthal's *r*, with values interpreted according to standard benchmarks (small: 0.10, medium: 0.30, large: 0.50) (Rosenthal 1994). Second, associations among all key variables (i.e., PD traits, interpersonal problems and mental health symptoms) were examined using Spearman's rho (*ρ*) correlation coefficient.

Third, a mediation model was tested to examine whether interpersonal problems mediated the relationship between PD traits and mental health symptoms. To operationalize the outcome variable, a composite mental health score was derived from the nine symptom dimensions of the SCL‐90 via principal component analysis. The first unrotated principal component, accounting for the largest proportion of shared variance, was retained and interpreted as a global index of mental health. In order to examine the role of PD traits at a higher‐order level, the 10 PDs assessed with the IPDE were grouped according to the DSM‐5 three‐cluster model: Cluster A (paranoid, schizoid, schizotypal), Cluster B (antisocial, borderline, histrionic and narcissistic) and Cluster C (avoidant, dependent and obsessive‐compulsive). Composite scores for each cluster were calculated by summing the respective item scores. Mediation analyses were conducted separately for each interpersonal problem using JASP to estimate indirect effects and their 95% confidence intervals (CIs). To control for potential confounding effects, age and gender were included as covariates in all mediation models.

## 2 Results

### 2.1 Gender Differences in PDs, Mental Health and Interpersonal Problems

Gender differences were observed in two domains. First, in the interpersonal problem related to self‐sacrificing, women scored higher than men (8.28 vs. 7.22). Second, significant differences were also found in narcissistic personality disorder, with men scoring slightly higher than women (*p* = 0.048). No gender differences were found in the remaining PDs or in mental health symptoms (see Table 1).

**TABLE 1:** Gender differences in personality disorders and interpersonal problems.

	Total sample (*N* = 291)	Males (*n* = 98)	Females (*n* = 193)	*p*	Rosenthal's *R*
Personality disorders					
Paranoid	2.35 (1.75)	2.34 (1.81)	2.37 (1.72)	0.767	0.017
Schizoid	2.78 (1.62)	2.54 (1.49)	2.93 (1.65)	0.081	0.102
Schizotypal	2.49 (1.99)	2.70 (2.12)	2.41 (1.92)	0.337	0.056
Histrionic	2.65 (1.85)	2.72 (1.90)	2.64 (1.83)	0.915	0.006
Antisocial	1.01 (1.31)	1.15 (1.44)	0.95 (1.24)	0.368	0.053
Narcissistic	2.13 (1.68)	2.41 (1.73)	2.01 (1.63)	0.048	0.116
Borderline	3.66 (2.28)	3.33 (2.19)	3.85 (2.30)	0.078	0.103
Obsessive‐compulsive	3.12 (1.78)	2.99 (1.80)	3.22 (1.75)	0.263	0.066
Dependent	2.71 (1.93)	2.52 (2.00)	2.83 (1.88)	0.105	0.095
Avoidant	3.70 (2.34)	3.60 (2.39)	3.76 (2.32)	0.549	0.035
Personality disorders clusters					
Cluster A	7.62 (4.30)	7.58 (4.43)	7.70 (4.22)	0.678	0.024
Cluster B	9.44 (5.41)	9.60 (5.46)	9.44 (5.35)	0.834	0.012
Cluster C	9.53 (4.42)	9.11 (4.43)	9.81 (4.35)	0.205	0.074
Interpersonal problems					
Domineering/controlling	2.36 (2.52)	2.32 (2.67)	2.39 (2.46)	0.513	0.038
Intrusive/needy	5.03 (3.55)	4.94 (3.72)	5.11 (3.47)	0.534	0.036
Self‐sacrificing	7.89 (4.17)	7.22 (4.18)	8.28 (4.10)	0.023	0.134
Overly accommodating	5.96 (3.35)	5.69 (3.22)	6.15 (3.39)	0.270	0.065
Nonassertive	5.24 (3.87)	5.03 (3.75)	5.37 (3.94)	0.500	0.040
Socially inhibited	3.97 (2.87)	3.84 (2.80)	4.06 (2.91)	0.470	0.042
Cold/distant	5.91 (3.04)	5.63 (2.92)	6.10 (3.06)	0.131	0.088
Vindictive/self‐centred	4.81 (3.18)	4.65 (3.45)	4.93 (3.04)	0.246	0.068
Mental health					
Depression	9.17 (3.30)	8.86 (3.11)	9.39 (3.32)	0.065	0.108
Anxiety	5.49 (2.91)	5.33 (2.92)	5.61 (2.90)	0.430	0.046
Hostility	2.56 (2.02)	2.74 (2.11)	2.50 (1.97)	0.427	0.047
Phobic anxiety	2.43 (2.13)	2.32 (2.02)	2.49 (2.19)	0.680	0.024
Somatization	5.96 (3.63)	5.50 (3.74)	6.23 (3.54)	0.102	0.096
Obsessive‐compulsive	6.38 (2.86)	6.64 (2.83)	6.30 (2.85)	0.309	0.060
Interpersonal susceptibility	4.81 (2.73)	4.92 (2.72)	4.80 (2.72)	0.757	0.018
Paranoid ideation	3.18 (2.08)	3.16 (2.02)	3.22 (2.09)	0.784	0.016
Psychoticism	3.52 (2.50)	3.68 (2.55)	3.47 (2.47)	0.568	0.034

### 2.2 Correlations Between PDs, Interpersonal Problems and Mental Health Problems

For Cluster A (i.e., paranoid, schizoid and schizotypal), significant associations were observed with all interpersonal problems except for intrusive/needy. Specifically, weak correlations emerged with the domineering/controlling, self‐sacrificing and vindictive/self‐centred subscales (*ρ*‐values ranging from 0.168 to 0.276), whereas moderate correlations were found with the remaining interpersonal difficulties (*ρ*‐values between 0.373 and 0.479). Regarding Cluster B (i.e., antisocial, borderline, histrionic and narcissistic), small correlations were observed with the self‐sacrificing, overly accommodating, nonassertive, socially inhibited and cold‐distant subscales (*ρ*‐values ranging from 0.119 to 0.268). A moderate correlation was found with the intrusive/needy subscale (*ρ* = 0.497), while strong correlations were observed with the domineering/controlling (*ρ* = 0.584) and vindictive/self‐centred (*ρ* = 0.551) subscales. For Cluster C (i.e., avoidant, dependent and obsessive‐compulsive), a small correlation was observed with the intrusive/needy subscale (*ρ* = 0.274). Moderate correlations were found with the domineering/controlling, self‐sacrificing, socially inhibited, cold‐distant and vindictive/self‐centred subscales (*ρ*‐values between 0.340 and 0.483). Strong associations were obtained for the overly accommodating (*ρ* = 0.540) and nonassertive (*ρ* = 0.500) subscales.

Finally, the global mental health score showed strong correlations with all personality clusters (*ρ*‐values ranging from 0.494 to 0.571), and with the nine interpersonal problems assessed (*ρ*‐values between 0.467 and 0.557) (see Table 2). For greater detail, Table S1 provides correlation matrices including each of the nine mental health subscales (i.e., Somatization, Obsession‐Compulsion, Interpersonal Sensitivity, Depression, Anxiety, Hostility, Phobic Anxiety, Paranoid Ideation and Psychoticism) and the 10 specific PDs (i.e., paranoid, schizoid, schizotypal, histrionic, narcissistic, borderline, antisocial, dependent, avoidant and obsessive‐compulsive).

**TABLE 2:** Spearman correlations between personality disorders (grouped by clusters), interpersonal problems and global mental health.

	1	2	3	4	5	6	7	8	9	10	11
Personality disorders											
1 Cluster A	—										
2 Cluster B	0.393**	—									
3 Cluster C	0.531**	0.395**	—								
Interpersonal problems											
4 Domineering/Controlling	0.276**	0.548**	0.342**	—							
5 Intrusive/Needy	0.055	0.497**	0.274**	0.523**	—						
6 Self‐sacrificing	0.168**	0.268**	0.361**	0.374**	0.718**	—					
7 Overly accommodating	0.479**	0.151**	0.540**	0.394**	0.440**	0.535**	—				
8 Nonassertive	0.429**	0.119*	0.500**	0.354**	0.439**	0.469**	0.752**	—			
9 Socially inhibited	0.426**	0.234**	0.483**	0.396**	0.538**	0.551**	0.751**	0.856**	—		
10 Cold/Distant	0.373**	0.254**	0.476**	0.428**	0.534**	0.632**	0.816**	0.699**	0.729**	—	
11 Vindictive/Self‐centred	0.173**	0.551**	0.340**	0.665**	0.852**	0.730**	0.472**	0.403**	0.510**	0.540**	—
12 Global mental health^a^	0.494**	0.501**	0.571**	0.499**	0.499**	0.467**	0.545**	0.535**	0.547**	0.557**	0.527**

* *p* ≤ 0.05.

** *p* ≤ 0.01.

^a^ Global mental health refers to composite score based on nine psychopathological symptoms (i.e., somatization, obsession‐compulsion, interpersonal sensitivity, depression, anxiety, hostility, phobic anxiety, paranoid ideation and psychoticism).

### 2.3 Mediating Role of Interpersonal Problems in the Relationship Between PDs and Global Mental Health

To examine whether interpersonal problems mediate the relationship between PDs and global mental health, eight separate mediation models (one for each interpersonal problem) were estimated, controlling for the effects of age and gender.

The a‐paths from the three personality clusters (Clusters A, B and C) to the eight interpersonal‐problem mediators were statistically significant in most of the models. Notwithstanding, four a‐paths failed to reach statistical significance: Cluster A to domineering/controlling (*β* = 0.027, SE = 0.038, 95% CI [−0.047, 0.102]); Cluster A to self‐sacrificing (*β* = −0.090, SE = 0.076, 95% CI [−0.239, 0.060]); Cluster B to socially inhibited (*β* = 0.044, SE = 0.033, 95% CI [−0.021, 0.109]); and Cluster B to cold/distant (*β* = 0.040, SE = 0.037, 95% CI [−0.032, 0.112]). Concerning the b‐paths, each interpersonal problem was significantly associated with poorer mental health in all models except the model in which domineering served as the mediator (*β* = 0.024, SE = 0.020, 95% CI [−0.016, 0.064]).

The indirect effects (i.e., the product of the a‐ and b‐paths) were statistically significant for most of the combinations, providing evidence that several interpersonal problems mediate the association between personality cluster scores and global mental health. However, a subset of indirect effects did not reach significance. Specifically, non‐significant indirect effects were observed for: (1) domineering/controlling in Clusters A, B and C (all *p*‐values ≥ 0.277); (2) self‐sacrificing in Cluster A (*p* = 0.268); and (3) socially inhibited (*p* = 0.213) and cold/distant (*p* = 0.289) in Cluster B.

**FIGURE 1:**
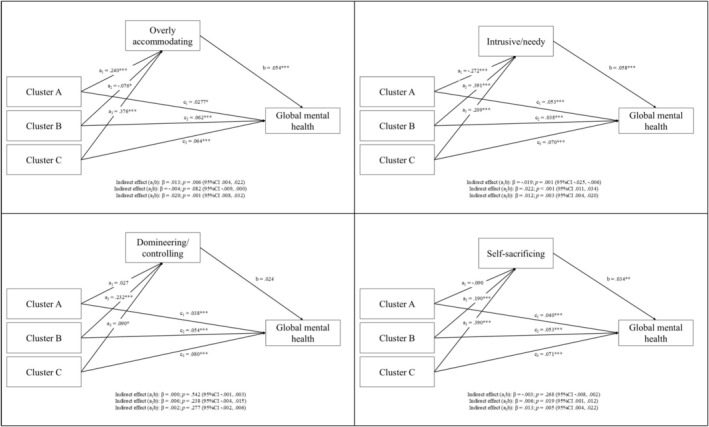
The mediating role of self‐sacrificing, overly accommodating, intrusive/needy and domineering/controlling in the relationship between personality disorders and mental health.

**FIGURE 2:**
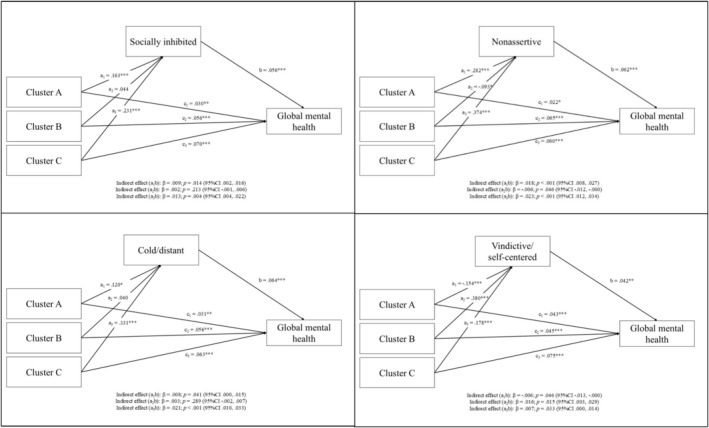
The mediating role of socially inhibited, nonassertive, cold/distant and vindictive/self‐centred in the relationship between personality disorders and mental health.

In summary, the pattern of results indicates that interpersonal problems mediate the association between personality cluster scores and mental health problems, although domineering/controlling and a small subset of cluster‐specific pathways did not support mediation in these models. Full path coefficients, *p*‐values and CIs for the indirect effects are reported in Figures 1 and 2.

## 3 Discussion

The primary objective of the present study was to analyse the interaction between PDs, interpersonal problems and global mental health in a clinical sample of individuals diagnosed with PDs, further exploring potential gender differences.

Regarding the first objective, the results reveal no gender differences in PD severity, with the sole exception of narcissistic PD. This specific difference aligns with meta‐analytic evidence indicating that men consistently exhibit higher levels of narcissism, particularly in facets related to entitlement and grandiosity (Grijalva et al. 2015). This disparity is frequently attributed to traditional gender socialization processes that encourage and reward dominance, competitiveness and authority in males (Grijalva et al. 2015; Weidmann et al. 2023). Beyond this specific exception, the overall lack of gender differences in the remaining PDs contrasts with a portion of the traditional epidemiological literature, which frequently reports differential diagnostic prevalence based on gender, noting a higher rate of antagonistic and eccentric spectrum disorders (such as Cluster A) in men, compared to a higher prevalence of conditions characterized by dependence, emotional lability and anxiety in women (APA 2022; Estévez et al. 2024; Grant et al. 2004). However, the results of this study align with contemporary research pointing to gender biases in the assessment and diagnosis of PDs, as well as in general clinical practice (Estévez et al. 2024; Observatorio de Salud, Sanidad y Farmacia (OdS) 2026). In this vein, a recent meta‐analysis by Huo et al. (2025) demonstrates that men with borderline personality meet the diagnostic criteria for borderline PD less frequently than women. Similarly, Jane et al. (2007) found that men and women with equivalent levels of symptomatology endorsed diagnostic items at different rates. These results could be explained by gender bias in the construction of diagnostic categories, which reflect stereotypes linked to traditional gender roles. For instance, studies have revealed that traits of impulsivity or emotional dysregulation are often interpreted primarily as externalizing disorders in men, whereas in women, they are largely coded as borderline or dependent disorders (Oredsson 2023; Skodol and Bender 2003). In other words, male emotional distress tends to be underdiagnosed or reinterpreted as behavioural and externalizing conflict, whereas female distress is frequently associated with internalizing pathology and tends to be overmedicalized. In fact, a recent study shows that women are prescribed more medication than men for the same diagnosis, and fewer complementary diagnostic tests are requested for them (Bozzatello et al. 2024; Obermeyer et al. 2019; Oroz et al. 2021; Qian et al. 2022; Ussher 2011).

Continuing with the first objective, the study results also show no significant gender differences in interpersonal problems, except for the self‐sacrificing subscale, where women score significantly higher than men. This result is consistent with gender socialization theories, which describe a significantly greater tendency among females to prioritize caring for others and maintaining affective bonds over their own needs (Emran et al. 2020; Maji and Dixit 2019). These findings again reveal women's tendency to adopt interpersonal styles that are more internalizing and submissive, characterized by personal renunciation to avoid bond rupture (Ghaed and Gallo 2006; Paris 2004). It is worth noting that studies such as that by Emran et al. (2020) indicate that the systematic inhibition of one's own needs to preserve the bond and relational harmony constitutes a high‐risk factor for mental health disorders, such as severe depressive symptomatology, anxiety and stress, somatization or eating disorders. Therefore, self‐sacrifice may operate as a relational vulnerability that aggravates the clinical picture in women.

The second objective was to study the relationship between personality clusters (A, B and C) and interpersonal problems. First, findings show that Cluster B disorders (i.e., antisocial, borderline, histrionic and narcissistic), characterized by traits such as impulsivity, emotional lability and rivalry, exhibited weak correlations with internalizing problems (i.e., self‐sacrifice, overly accommodating, social inhibition or coldness); moderate correlations with the intrusive/needy dimension, which may reflect the lability and urgent affective demand typical of borderline PD; and high correlations with the domineering/controlling and vindictive/self‐centred dimensions. This finding reinforces the literature indicating that individuals in Cluster B present relational conflicts predominantly through antagonism and hostility, coercive and dominant strategies, externalization of behaviours or the transgression of others' boundaries (Hopwood et al. 2018; Ociskova et al. 2023; Oliva et al. 2023; Wilson et al. 2017).

In contrast, Cluster C disorders (i.e., avoidant, dependent and obsessive‐compulsive), characterized by anxiety and avoidance, showed weak correlations with the intrusive/dependent styles; and moderate correlations with the domineering/controlling, self‐sacrificing, socially inhibited, cold/distant and vindictive/self‐centred types. In this context, the moderate correlations with the control domain might reflect the perfectionism typical of obsessive‐compulsive traits; the moderate correlations with the domains of egocentrism, social inhibition or coldness could reflect avoidant personality traits, while self‐sacrifice could reflect dependent traits. Finally, strong associations were found between Cluster C and the overly accommodating relational pattern and lack of assertiveness. These findings reveal an inhibited interpersonal style, largely directed at avoiding relational conflicts at the expense of one's own needs and marked by fear of rejection (Estévez et al. 2025; Hopwood et al. 2018; Momeñe et al. 2022).

Regarding Cluster A (i.e., paranoid, schizoid and schizotypal), which is marked by detachment and eccentricity, significant associations were observed with almost all dimensions of the IIP, except for the intrusive/dependent style. This result aligns with previous evidence, as the core of these PDs lies in social alienation, interpersonal anhedonia and relational mistrust, which distances them from developing active proximity‐seeking and relational dependence dynamics (d'Huart et al. 2023). Furthermore, the weak association with the dimensions of dominance, self‐sacrifice or egocentrism suggests that the relational difficulties of Cluster A do not centre on power dynamics or caring for others, but rather on severe difficulties in establishing secure, warm and functional connections with the environment (Pincus and Wright 2011; Wright et al. 2012).

The third and final objective of the study was to examine whether interpersonal problems mediate the relationship between PDs and mental health. The findings of this study confirm that interpersonal problems act as a mediating mechanism between PDs and mental health problems. These results may be of great clinical relevance, as they suggest that interpersonal difficulties and the lack of functionality in the social sphere could operate as a vehicle for PDs to manifest as mental health problems. Therefore, impairment in the social sphere could help explain the comorbidity between PDs and psychiatric symptomatology.

However, not all mediation models in the present study were significant. Most notably, the domineering/controlling style was the only interpersonal problem not associated with a worsening of general mental health. This absence of mediation, consistent across all three clusters, underscores the relational nature of interpersonal styles based on coercion and dominance. Typically, these types of dynamics are egosyntonic and generate profound suffering in the environment but tend to protect the perpetrator from experiencing acute internalizing symptomatology, such as anxiety or depression (Hopwood et al. 2018; Pincus et al. 2009).

On the other hand, self‐sacrifice did not mediate mental health problems in Cluster A (paranoid, schizoid and schizotypal). These findings are in line with previous theoretical models (Fanti et al. 2023; Freeman and Loe 2023), as suspiciousness, mistrust and social distancing typical of Cluster A PDs are not characterized by personal renunciation in favour of bonding. Similarly, social inhibition and cold/distant did not act as mediators for Cluster B (antisocial, borderline, histrionic and narcissistic), which empirically reinforces theoretical models suggesting that the personality profiles of this cluster have externalizing, impulsive attachment tendencies based on attention‐seeking, distancing them from the dynamics of social withdrawal (Leichsenring et al. 2023; Wang et al. 2023; Young et al. 2003).

### 3.1 Limitations

The results of the present study must be interpreted in light of several limitations. First, the cross‐sectional nature of the research design precludes establishing causal relationships between PDs, interpersonal problems and mental health deterioration. Second, the sample included a very broad age range (18–76 years), although analyses showed that age did not significantly alter the mediation models. Third, data collection spanned a substantial time period (from 2010 to 2021). Although such an extended timeframe is often necessary to recruit a specialized clinical sample, potential cohort effects cannot be entirely ruled out. Importantly, social, economic, contextual and health‐related events (e.g., COVID‐19 pandemic) may have affected variables and should be considered when interpreting the findings. Fourth, the assessment was conducted using self‐report instruments, which are inherently subject to potential social desirability biases or introspection deficits. Fifth, as this is a strictly clinical sample, the observed profiles and severity may differ significantly from those observed in the general population or other healthcare settings. Additionally, there is an imbalance in gender distribution, with an overrepresentation of women (*n* = 193) compared to men (*n* = 98). Although this asymmetry reflects real‐world demographic rates of treatment‐seeking behaviour in mental health consultations (Alanazi et al. 2025; Nam et al. 2010), it is necessary to replicate these findings in more gender‐balanced samples. Finally, the present study did not account for several potentially confounding clinical or psychosocial variables. Broader psychosocial dimensions, including perceived social support, socio‐occupational adjustment, emotion regulation and mentalization or attachment styles, for example, were not included in the analyses. Future studies should incorporate these covariates to provide a more comprehensive understanding of the interplay between PDs, interpersonal problems and mental health.

### 3.2 Conclusion and Clinical Implications

In conclusion, the results of the present study indicate that interpersonal problems act as a significant mediating factor in the relationship between PDs and overall mental health problems. Similarly, regarding intervention for patients diagnosed with a PD, these findings suggest that a treatment objective should be addressing relational styles and interpersonal conflicts. This is paramount given that relational functioning appears to be closely linked to the exacerbation of mental health problems and the overall severity of the PDs. Therefore, therapeutic frameworks that explicitly target interpersonal problems are highly recommended. Evidence‐based interventions such as Dialectical Behaviour Therapy (DBT), which incorporate specific modules for interpersonal effectiveness, Mentalization‐Based Treatment (MBT), Schema Therapy, mindfulness or Cognitive‐Analytic Therapy could be considered as valuable options (Bateman and Fonagy 2016; Bilbao et al. 2016; Linehan 2015; Mirapeix 2015; Stoffers‐Winterling et al. 2022; Young et al. 2003). These approaches aim to equip patients with the necessary skills to navigate social conflicts, improve emotional regulation within relationships and ultimately help alleviate the mental health problems associated with PD traits.

## Funding

The authors have nothing to report.

## Disclosure

The authors of this article declare that they did not use AI for any part of the study, nor for the writing of the article.

## Ethics Statement

The study was conducted in accordance with the Declaration of Helsinki. All participants provided informed consent before completing the questionnaires and were assured of confidentiality and anonymity regarding their responses. They were informed that participation was entirely voluntary, with no compensation provided.

## Conflicts of Interest

The authors declare no conflicts of interest.

## Supporting information


**Table S1:** Spearman correlations between personality disorders, interpersonal problems and mental health.

## Data Availability

The datasets generated during and/or analysed during the current study are not publicly available due to confidentiality.
