# Supplementary material for: Interpersonal Problems as Mediators of the Association Between Personality Disorders and Mental Health
Source: Clin Psychol Psychother. 2026 Jul 16;33(4):e70307. doi: 10.1002/cpp.70307 (PMC13374870; doi:10.1002/cpp.70307)
Supplement: Supplementary file 1 — Table S1: Spearman correlations between personality disorders, interpersonal problems and mental health. [file CPP-33-e70307-s001.docx]

**Table S1**

*Spearman correlations between personality disorders, interpersonal problems and mental health.*

|  | 1 | 2 | 3 | 4 | 5 | 6 | 7 | 8 | 9 | 10 | 11 | 12 | 13 | 14 | 15 | 16 | 17 | 18 | 19 | 20 | 21 | 22 | 23 | 24 | 25 | 26 |
| --- | --- | --- | --- | --- | --- | --- | --- | --- | --- | --- | --- | --- | --- | --- | --- | --- | --- | --- | --- | --- | --- | --- | --- | --- | --- | --- |
| Personality disorders |  |  |  |  |  |  |  |  |  |  |  |  |  |  |  |  |  |  |  |  |  |  |  |  |  |  |
| 1. Paranoid |  |  |  |  |  |  |  |  |  |  |  |  |  |  |  |  |  |  |  |  |  |  |  |  |  |  |
| 1. Schizoid | .296** |  |  |  |  |  |  |  |  |  |  |  |  |  |  |  |  |  |  |  |  |  |  |  |  |  |
| 1. Schizotypal | .503** | .453** |  |  |  |  |  |  |  |  |  |  |  |  |  |  |  |  |  |  |  |  |  |  |  |  |
| 1. Histrionic | .266** | -.071 | .215** |  |  |  |  |  |  |  |  |  |  |  |  |  |  |  |  |  |  |  |  |  |  |  |
| 1. Antisocial | .359** | .041 | .322** | .426** |  |  |  |  |  |  |  |  |  |  |  |  |  |  |  |  |  |  |  |  |  |  |
| 1. Narcissistic | .252** | -.020 | .149** | .386** | .343** |  |  |  |  |  |  |  |  |  |  |  |  |  |  |  |  |  |  |  |  |  |
| 1. Borderline | .542** | .203** | .429** | .525** | .428** | .297** |  |  |  |  |  |  |  |  |  |  |  |  |  |  |  |  |  |  |  |  |
| 1. Obsessive-compulsive | .285** | .288** | .181** | .102* | .092 | .249** | .256** |  |  |  |  |  |  |  |  |  |  |  |  |  |  |  |  |  |  |  |
| 1. Dependent | .297** | .124** | .308** | .396** | .209** | .129** | .444** | .186** |  |  |  |  |  |  |  |  |  |  |  |  |  |  |  |  |  |  |
| 1. Avoidant | .433** | .456** | .474** | .123** | .119* | .076 | .385** | .317** | .407** |  |  |  |  |  |  |  |  |  |  |  |  |  |  |  |  |  |
| Interpersonal problems |  |  |  |  |  |  |  |  |  |  |  |  |  |  |  |  |  |  |  |  |  |  |  |  |  |  |
| 1. Domineering/ controlling | .319** | .032 | .298** | .407** | .407** | .290** | .497** | .304** | .252** | .224** |  |  |  |  |  |  |  |  |  |  |  |  |  |  |  |  |
| 1. Intrusive/needy | .096 | -.055 | .118* | .541** | .237** | .294** | .419** | .190** | .397** | .071 | .523** |  |  |  |  |  |  |  |  |  |  |  |  |  |  |  |
| 1. Self-sacrificing | .130* | .123* | .178** | .268** | .052 | .121* | .332** | .231** | .410** | .196** | .374** | .718** |  |  |  |  |  |  |  |  |  |  |  |  |  |  |
| 1. Overly accommodating | .316** | .444** | .418** | .001 | .098 | .018 | .326** | .253** | .314** | .590** | .394** | .440** | .535** |  |  |  |  |  |  |  |  |  |  |  |  |  |
| 1. Nonassertive | .267** | .411** | .386** | .030 | .039 | .002 | .259** | .141* | .325** | .579** | .354** | .439** | .469** | .752** |  |  |  |  |  |  |  |  |  |  |  |  |
| 1. Socially inhibited | .280** | .363** | .414** | .143* | .120* | .080 | .358** | .168** | .414** | .469** | .396** | .538** | .551** | .751** | .856** |  |  |  |  |  |  |  |  |  |  |  |
| 1. Cold/distant | .252** | .293** | .370** | .177** | .127* | .112 | .337** | .248** | .338** | .449** | .428** | .534** | .632** | .816** | .699** | .729** |  |  |  |  |  |  |  |  |  |  |
| 1. Vindictive/self-centered | .183** | .008 | .230** | .516** | .321** | .321** | .474** | .241** | .415** | .145* | .665** | .852** | .730** | .472** | .403** | .510** | .540** |  |  |  |  |  |  |  |  |  |
| Mental health |  |  |  |  |  |  |  |  |  |  |  |  |  |  |  |  |  |  |  |  |  |  |  |  |  |  |
| 1. Somatization | .318** | .177** | .235** | .244** | .232** | .146** | .381** | .320** | .260** | .244** | .375** | .370** | .402** | .370** | .357** | .328** | .368** | .395** |  |  |  |  |  |  |  |  |
| 1. Obsession-compulsion | .343** | .211** | .278** | .266** | .184** | .153** | .384** | .321** | .360** | .379** | .353** | .435** | .428** | .423** | .454** | .420** | .437** | .438** | .629** |  |  |  |  |  |  |  |
| 1. Interpersonal sensitivity | .477** | .318** | .422** | .252** | .278** | .138** | .465** | .235** | .406** | .572** | .447** | .427** | .372** | .615** | .615** | .597** | .573** | .458** | .512** | .693** |  |  |  |  |  |  |
| 1. Depression | .427** | .311** | .318** | .271** | .174** | .134** | .501** | .249** | .445** | .442** | .341** | .441** | .472** | .542** | .525** | .547** | .533** | .437** | .610** | .689** | .699** |  |  |  |  |  |
| 1. Anxiety | .337** | .162** | .240** | .284** | .235** | .144** | .445** | .268** | .399** | .365** | .424** | .449** | .437** | .436** | .417** | .417** | .472** | .453** | .726** | .665** | .602** | .704** |  |  |  |  |
| 1. Hostility | .485** | .153** | .417** | .364** | .439** | .230** | .590** | .224** | .272** | .319** | .579** | .404** | .313** | .392** | .394** | .449** | .414** | .465** | .501** | .530** | .639** | .552** | .566** |  |  |  |
| 1. Phobic anxiety | .204** | .125* | .153** | .180** | .125* | .073 | .298** | .152** | .357** | .349** | .333** | .364** | .350** | .415** | .450** | .390** | .415** | .367** | .531** | .534** | .517** | .522** | .678** | .356** |  |  |
| 1. Paranoid ideation | .567** | .265** | .454** | .304** | .352** | .258** | .492** | .248** | .384** | .457** | .416** | .429** | .401** | .470** | .430** | .466** | .484** | .457** | .497** | .660** | .782** | .646** | .536** | .618** | .384** |  |
| 1. Psychoticism | .401** | .241** | .363** | .344** | .294** | .267** | .528** | .290** | .456** | .451** | .442** | .483** | .453** | .506** | .475** | .539** | .561** | .509** | .564** | .705** | .736** | .729** | .683** | .555** | .542** | .733** |
| *Note*. * *p* ≤ .05. ** *p* ≤ .01. | | | | | | | | | | | | | | | | | | | | | | | | | | |
